# **Studies on the mitochondrial, immunological and inflammatory effects of solvent fractions of Diospyros mespiliformis** Hochst **in Plasmodium berghei-infected mice**

**DOI:** 10.1038/s41598-021-85790-6

**Published:** 2021-03-25

**Authors:** Oluwole Moses David, John Oludele Olanlokun, Bisola Evelyn Owoniyi, MoyinOluwa Ayeni, Oluwakemi Ebenezer, Neil Anthony Koorbanally

**Affiliations:** 1 Department of Microbiology, Ekiti State University, Ado-Ekiti, Nigeria; 2 Laboratories for Biomembrane Research and Biotechnology, Department of Biochemistry, College of Medicine, University of Ibadan, Ibadan, Nigeria; 3 School of Chemistry and Physics, University of KwaZulu-Natal, Durban, South Africa

**Keywords:** Biochemistry, Cell biology, Drug discovery, Immunology

## Abstract

The use of medicinal plants in the treatment of malaria is gaining global attention due to their efficacy and cost effectiveness. This study evaluated the bioactivity-guided antiplasmodial efficacy and immunomodulatory effects of solvent fractions of *Diospyros mespiliformis* in mice infected with a susceptible strain of *Plasmodium berghei* (NK 65). The crude methanol extract of the stem of *D. mespiliformis* (DM) was partitioned between *n*-hexane, dichloromethane, ethyl acetate and methanol. Male Swiss mice (20 ± 2 g) infected with *P. berghei* were grouped and treated with vehicle (10 mL/kg, control), Artemether lumefantrine (10 mg/kg), 100, 200 and 400 mg/kg of *n*-hexane, dichloromethane, ethyl acetate and methanol fractions of *D. mespiliformis* for seven days. Blood was obtained for heme and hemozoin contents while serum was obtained for inflammatory cytokines and immunoglobulins G and M assessments. Liver mitochondria were isolated for mitochondrial permeability transition (mPT), mitochondrial F_1_F_0_ ATPase (mATPase) and lipid peroxidation (mLPO) assays. The GC–MS was used to identify the compounds present in the most potent fraction. The dichloromethane fraction had the highest parasite clearance and improved hematological indices relative to the drug control. The heme values increased, while the hemozoin content significantly (*P* < 0.05) decreased compared with the drug control. The highest dose of HF and MF opened the mPT pore while the reversal effects of DF on mPT, mATPase and mLPO were dose-dependent. The levels of IgG, IgM and TNFα in the DF group were significantly higher than the drug control, while the IL-1*β* and IL-6 values did not vary linearly with the dose. Lupeol and Stigmastan-3,5-diene were the most abundant phytochemicals in the DF. The outcome of this study showed that the DF has immunomodulatory effects in infected mice, reduced proliferation of the malaria parasite and thus protect liver cells.

## Introduction

Malaria is a major life-threatening human parasitic infection, especially in sub-Saharan Africa^1,2^. Despite all interventions to reduce the incidence of malaria, the rate of mortality and morbidity keep increasing due to treatment failures and aetiologic agents are becoming super bugs with high resistance to drugs, especially orthodox drugs^3–5^. *Plasmodium* species are responsible for four out of every five cases of malaria infections and could lead to death of an infected person within hours to a few days after infection^6^. It could also cause a chronic infection that could last for years^7,8^.

Medicinal plants have been used for the treatment of human diseases for ages and has offered help for the treatment and management of various diseases. For this reason, quite a number of medicinal plants have been screened for their treatment purpose and documented in pharmacopoeias^9^. *Diospyros mespiliformis* Hochst belongs to the family *Ebenaceae*. It is an evergreen tree that has been used in the treatment of different human diseases^10,11^. The extract of the plant has been reported to have good antioxidant, anti-proliferative and antimicrobial activities^12–14^. Previous studies^15,16^ have justified the indigenous claim for the use of *D. mespiliformis* in the treatment of malaria. Although, *D. mespiliformis* and other medicinal plants have been used for the management of uncomplicated and resistant malaria (caused by drug resistant strains of *Plasmodium* parasites), the mechanism of action by which *D. mespiliformis* combats malaria has not been explored. Although parasites have evolved quite a number of ways to develop resistance against drugs as their own means of adaptation and survival, the innate immune system of the host has been developed to overcome this evading strategy of the pathogens. With a specific immune response system, the host gets rid of many pathogens and sometimes the mechanism of action of some drugs relies on their effectiveness to boost the host immune response^17^.

Increase in severe parasitic infections in immunocompromised patients have increased the interest of researchers in antimalarial drug development and the possible mechanism of action of identified drugs. The interaction of anti-parasitic drugs with the immune system has long been discovered. Orthodox antimalarial drugs such as chloroquine, pyrimethamine and quinacrine have been found to depress human polymorphonuclear neutrophils^18^. The antimalarial mechanism of action of some drugs have been proposed to involve their effects on the immune system, which include: interference with lysosomal acidification, inhibition of proteolysis and antigen presentation, modulation of cytokine production and inhibition of matrix metalloproteinases^19^.

Artemisinins have been reported to have immunomodulatory properties such as inflammation, autoimmunity, and delayed-type hypersensitivity^20^. Specifically, they have been found to decrease the secretion of pro-inflammatory cytokines and enhance the release of Tumor Necrosis Factor (TNFα), and IL-1*β*^21^. It follows then that medicinal plants with antimalarial properties may stimulate the immune system to elicit their antimalarial potency through a similar mechanism. Previous studies have shown that the healing effect observed with patients using plant decoctions could partly be due to their stimulating activity of the immune system^22^. In this regard, pectic polysaccharides isolated from the leaves of *Trichilia emetica* (*Meliaceae*), a plant used in traditional medicine in Mali, has been shown to activate the complement system and induce the proliferation of T and B-lymphocytes^23^.

Although, the antiplasmodial effect of the crude methanol extract of *D. mespiliformis* has been substantiated^24^, the activity-guided assay of its immunomodulatory effect and probable mechanism of action have not been reported. It is against this background that we reported the activity-guided assay of various solvent fractions of *D. mespiliformis* against malaria caused by *P. berghei*, the immunomodulatory and molecular mechanisms of its anti-inflammatory and mitochondrial dysfunction potentials in mice*.*

## Materials and methods

### Collection and extraction of plant materials

The woody stem of *D. mespiliformis* was obtained fresh from a medicinal herb agent at the Oje Market, Ibadan, and authenticated at Ekiti State University Herbarium. A voucher (specimen number: UHAE2017/062) was deposited in the University Herbarium. The plant material was air dried, chopped into small pieces and ground into powder. It was later sieved and 400 g of the powder was soaked in 1 L absolute methanol with occasional shaking using a mechanical shaker (GFL, Germany) for 72 h. The mixture was then filtered through Whatman No. 1 filter paper and concentrated under reduced pressure using a rotary evaporator (Stuat, United Kingdom). The concentrated methanol extract was pre-adsorbed on Thin Layer Chromatography (TLC) silica gel and subject to Vacuum Liquid Chromatography. The column was eluted successively using *n*-hexane, dichloromethane, ethyl acetate and methanol. The filtrate obtained was concentrated and evaporated to dryness using a water bath at 40 °C to obtain *n*-hexane (HF), dichloromethane (DF), ethyl acetate (EF) and methanol (MF) fractions, respectively.

### Source and grouping of experimental animals

A total of seventy male Swiss mice (20 ± 2) g that were 3 weeks old were obtained from the Institute of Advanced Medical Research Training (IAMRAT), College of Medicine, University of Ibadan, Nigeria. The animals were acclimatized for one week and infected intraperitoneally with infected erythrocytes (inoculum size = X10^7^) from a donor mouse. The appropriate volume of infected blood from the donor mouse used to infect experimental Swiss mice was determined using a hemocytometer. Parasitemia was confirmed after 72 h and the animals were randomly assigned to twelve groups of five animals each as follows: five animals each were assigned to the positive control group (treated with 10 mg/kg Artemether Lumefantrin) and, negative control group (treated with 10 mL/kg vehicle, 10% dimethyl sulfoxide (DMSO)). Other groups were treated with graded doses (100, 200 and 400 mg/kg) of HF, DF, EF and MF, prepared by dissolving each fraction in 10% DMSO respectively for 7 days. The 10% DMSO was prepared by dissolving 10 mL DMSO in 90 mL of solution.

### Ethical consideration

All animals used for this experiment were handled in accordance with the rules and regulations for management of animals used in research as contained in the Guide for the Care and Use of Laboratory Animals^25^. Furthermore, this study was approved by the Ekiti State University Office of Research and Development, and an Approval Number ORD/AD/EAC/19/0057 assigned to the study.

### Determination of percentage parasitemia and parasite clearance

Ryley and Peters’ method of established infection was used for the antiplasmodial assay with slight modification^26^. The modification here was that unlike in the adopted method where treatment lasted for 5 days with daily (24 h) assessment of parasitemia, ours was at two days (48 h) interval. This is because we used plant extract and since the concentration of the active principle per unit volume of the fractions of *D. mespiliformis* taken may be small to elicit immediate antiplasmodial activity, compared to orthodox drugs, we may not observe significant decrease in the parasite load within 24 h. Thus, a 5 day treatment plan with 48 h interval for the assessment of parasitemia was adopted. Malaria parasites were detected in the blood after 72 h of inoculation (day 0) and treatment commenced the same day parasites were detected in the blood (which is still day 0). To assess parasite load in the blood or the potency of the administered solvent fractions of *D. mespiliformis*, blood was obtained from the tail region of the individual mouse and a thin film smear was made on the slides, air-dried and fixed in absolute methanol. Thereafter, the slides were stained with 10% Giemsa stain for 30 min, rinsed with buffered water and air-dried. Slides were collected at two day intervals. The percentage parasitemia and parasite clearance were determined by microscopy, counting infected and normal red blood cells on each slide using 3 different fields at random. Infected and uninfected erythrocytes were counted; percentage parasitemia and parasite clearance were calculated as follows:

$${\text{Percentage}}\,{\text{Parasitemia = }}\frac{{{\text{Number}}\,{\text{of}}\,{\text{infected}}\,{\text{erythrocytes}}\,{\text{counted}}}}{{{\text{Total}}\,{\text{erythrocytes}}\,\left( {{\text{infected}}\,{\text{and}}\,{\text{non - infected}}} \right)}} \times 100$$

$${\text{Percentage}}\,{\text{Clearance}} = \frac{{{\text{Counted}}\,{\text{infected}}\,{\text{erythrocytes}}\,\left( {{\text{control}}} \right){-}{\text{Counted}}\,{\text{infected}}\,{\text{erythrocytes}}\,\left( {{\text{Test}}} \right)}}{{{\text{Counted}}\,{\text{infected}}\,{\text{erythrocytes}}\,\left( {{\text{control}}} \right)}} \times 100$$

### Assessment of heme and hemozoin contents

The total heme content was estimated from blood withdrawn from experimental animals^27^. Briefly, 10 µL blood was mixed with 250 µL 10% SDS (w/v) and 250 µL 1 M NaOH, followed by sonication for 10 min. The tubes were incubated for 2 h at room temperature and the total heme content determined on a spectrophotometer at 404 nm. Assuming the molar absorption coefficient of heme to be 9.08 × 10^4^/M/cm, the concentration was expressed as mmol of heme/mL of blood.

To determine the hemozoin content, 10 µL blood was completely lysed using 0.08% saponin and centrifuged in an ultracentrifuge (Thermo scientific, USA) at 2000 g. The supernatant was discarded and the pellet washed (three times) with 250 µL of 25% SDS buffered (pH 7.4) with 25 mM Tris–HCl. The pellet was incubated at 37 °C for 24 h in the buffer. After incubation, the tubes were centrifuged at 2000 g and the pellet hydrolyzed using 1 M NaOH. The absorbance was read at 404 nm. The hemozoin content was estimated and expressed as µmol heme/ mL of blood^28^.

### Isolation of mouse liver mitochondria

Low-ionic-strength liver mitochondria were isolated from treated mice according to the previously described method by Johnson and Lardy^29^. The animals were sacrificed by cervical dislocation, dissected and the liver excised, mopped and weighed. The liver was washed several times in isolation buffer (210 mM Mannitol, 70 mM Sucrose, 5 mM 4-(2-hydroxymethyl)ethanesulfonic acid basified with potassium hydroxide (Hepes–KOH, pH 7.4) and 1 mM ethylene glycol-bis(*β*-aminoethyl ether)-*N*,*N*,*N'*,*N'*-tetraacetic acid) (EGTA) to remove blood stain. Thereafter, a 10% suspension was homogenized and the homogenate loaded into a cold (4 °C) centrifuge (Sigma 300-K, Germany) and spun at 260 g twice for 5 min each time. The supernatant was centrifuged again at 1500 g to pellet mitochondria, which was later washed with washing buffer (210 mM Mannitol, 70 mM Sucrose, 5 mM Hepes–KOH (pH 7.4) and 0.5% BSA) twice at 1400 g for 10 min each time. Washed mitochondria were dispensed in aliquots into Eppendoff tubes using suspension buffer (210 mM Mannitol, 70 mM Sucrose, 5 mM Hepes–KOH (pH 7.4). Isolation of mitochondria used for F_1_F_0_ ATPase activity assay followed similar steps explained above using 0.25 M sucrose buffer only.

### Protein determination

Mitochondrial protein was estimated according to the method of Lowry et al.^30^ using Bovine Serum Albumin (BSA) as standard. Ten (10) µL mitochondria was added to 990 µL distilled water in test tubes and 3 mL reagent D (100:1:1 combination of: 2 g Na_2_CO_3_ in 0.1 M NaOH, 2% K-Na tatrate and 1% CuSO_4_) was later added. This was mixed and allowed to stand at room temperature (28 °C) for 10 min. Folin–Ciocalteau (0.3 mL of a five-fold dilution) was later added and the mixture was left for 30 min, vortexed and the absorbance read at 750 nm on a Camspec M105 spectrophotometer.

### Assessment of mitochondrial membrane permeability transition in mouse liver mitochondria

The mitochondrial permeability transition was monitored as previously described by Lapidus and Sokolove^31^ with slight modification. Mitochondrial protein (0.4 mg/mL) from un-infected animals were incubated in adequate suspension buffer containing 8 µM rotenone for 3 min. Thirty seconds later, 5 mM succinate was added and the absorbance monitored at 540 nm for 12 min at 30 s intervals. To assess mitochondrial susceptibility to calcium induction of pore opening, similar mitochondrial protein was incubated in suspension buffer-rotenone mixture for 3 min, after which 3 µM calcium chloride (CaCl_2_) was added and 30 s later 5 mM succinate added, and the absorbance measured. Spermine inhibition of calcium-induced opening was carried out by incubating mitochondrial protein in suspension buffer-rotenone mixture containing 4 mM spermine for 3 min and 30 s later, succinate was added and absorbance measured. Isolated mitochondria susceptible to calcium induction of mitochondrial permeability transition and successfully reversed by spermine are adjudged uncoupled, viable and suitable for the study. Corresponding mitochondrial proteins from isolated hepatic mitochondria of treated groups were similarly treated in the absence of calcium and the pore opening compared with the normal, un-infected control.

### Mitochondrial F_1_F_0_ ATPase activity

Sucrose (25 mM), KCl (0.5 mM), 65 mM Tris(hydroxymethyl)aminomethane acidified with hydrochloric acid (Tris–HCl, pH 7.4), were added to all test tubes in triplicate. Mitochondrial proteins (0.5 mg/mL) were added to designated tubes and transferred to a shaker water bath at 25 °C. Adenosinetriphosphate (ATP, 1 mM) was added to the tubes while Uncoupler (25 µM of 2,4-dinitrophenol) was added to the designated tubes. The whole volume was uniformly made up and the test tubes incubated for 30 min. Sodium dodecysulfate, (SDS, 10%) was added after incubation to stop the reaction. One mL of the reaction mixture in each tube was withdrawn into separate test tubes and 1 mL of 1.25% ammonium molybdate (prepared in 6.5% H_2_SO_4_) and 1 mL of 9% freshly prepared ascorbic acid sequentially added. This was allowed to stand for 30 min, vortexed, and the absorbance read at 660 nm against reagent blank^32^.

### Assay of mitochondrial lipid peroxidation

A modified thiobarbituric acid reactive substance (TBARS) assay was used to measure the extent of lipid peroxidation. The mitochondrial test sample was mixed with 1.6 mL Tris basified with potassium chloride (Tris-KCL) buffer to which 0.5 mL 30% trichloro acetic acid was added. Then 0.5 mL of 0.75% thiobarbituric acid was added, and the test tubes were placed in a water bath at 80 °C for 45 min. This was then cooled on ice to room temperature (28 °C) and centrifuged at 400 g for 10 min. The clear supernatant was collected and the absorbance measured against a reference blank of distilled water^33,34^. The TBARS level was calculated using an extinction co-efficient of 0.156/µM/cm.

$${\text{Lipid}}\,{\text{Peroxidation}}\,\left( {{\text{nmole}}\,{\text{TBARS}}/{\text{mg}}\,{\text{protein}}} \right) \, = \frac{Absorbance \,x\, Volume\, of\, mixture}{{E_{532nm} \, x\, Volume\, of\, Sample\, x\, mgprotein/ml}}$$

### Measurement of hematological parameters

#### Packed cell volume

Packed Cell Volume (PCV) was determined as previously described^35^. Blood samples were aspirated into non-heparinized tubes, sealed with plasticin and centrifuged in hematocrit centrifuge at 250 g for 5 min. The PCV was read using a hematocrit reader.

#### Total white blood cell count (WBC)

Total WBC was determined as previously described^35^. Whole blood sample (20 μL) was added to 380 μL of Turk solution, mixed and allowed to settle for 2 min. The samples were thereafter loaded into Newbauer counting chamber and put inside a humid chamber for 1 min. It was then observed under the microscope using a × 10 objective.

#### Platelet count

Whole blood sample (20 μL) was added to 380 μL of an ammonium oxalate solution, allowed to settle for 2 min and then loaded into a counting chamber, and put inside a humid chamber for 1 min. Counting was done using a × 40 objective.

### Differential WBC count for lymphocytes, monocytes, eosinophils and neutrophils

Differential WBC count was determined as previously described^34^. A thin film of whole blood was made on a grease-free slide, air-dried and stained with Leishman stain for 5 min. The stain was thereafter diluted on the slide with phosphate buffered saline and the slide was further allowed to be stained for 10 min, rinsed, mopped, air-dried and read under a microscope using an oil immersion objective.

### Assay of immunological changes in the experimental animals

Serum was obtained from whole blood collected from the heart with the use of a 1 mL syringe, aspirated into plain sample bottles and allowed to clot. The clotted blood was spun in the centrifuge at 400 g for 5 min. The serum was aspirated into another clean plain sample bottle using a pasteur pipette. The levels of immunoglobulin M (IgM) and immunoglobulin G (IgG) were determined in the serum of the experimental animals treated with dichloromethane fraction of *D. mespiliformis* according to the kits manufacturers’ protocols (Fortress Diagnostics, Antrim, U.K). ELISA Kits (Elabscience, USA) were used to determine the levels of IL-6 (Interleukin 6), IL-1*β* and tumour necrosis factor (TNF-*α*). In all the immunological tests, the manufacturer’s procedures were strictly followed.

### Gas chromatography-mass spectrometry (GC–MS) analysis of the dichloromethane fraction

The probable phytochemicals present in the dichloromethane fraction of *D. mespiliformis* likely responsible for its biological action were determined using Gas Chromatography (USA) with a coupled mass detector. The GC column analyzer has an injector set at 200 °C including an ion source temperature at 250 °C. The carrier gas was helium set at a flow rate of 1 mL/min. Essential components of the dichloromethane fraction of *D. mespiliformis* were detected depending on their retention times and mass fragmentation patterns that had good matches with compounds in the NIST library software.

### Statistical analysis

The experiments were conducted in triplicate and repeated twice. Representative profiles of absorbance of mitochondria were used for the mitochondrial permeability transition assays. For other assays, data were expressed as mean ± standard deviation of triplicate readings. Ungrouped data from this study were analyzed using One-way ANOVA followed by Tukey’s post hoc comparison among data in columns while grouped data were analyzed using two-way ANOVA followed by Dunnett’s post hoc multiple comparison test using GraphPad prism 7.0. The level of significance was set at *P* < 0.05.

### Ethics approval

This study was approved by the Ekiti State University Office of Research and Development ethics committee review board and an Approval Number ORD/AD/EAC/19/0057 assigned to the study. Furthermore, this study was carried out in compliance with the ARRIVE guidelines and in accordance with relevant guidelines and regulations.

### Consent for publication

All authors consent to the publication of this article.

## Results

### Dichloromethane fraction of *D. mespiliformis* decreases parasite load and increases parasite clearance

The comparative results of the efficacy of various solvent fractions of *D. mespiliformis* against the malaria parasite using a decrease in parasite load and extent of parasite clearance is reported in Table 1a and b. Specifically, on day 1 all doses of fractions of *D. mespiliformis* exert varying decreases in parasite level corresponding to their antimalarial efficacy. Only the parasite levels of 200 mg/kg of DF, 100 and 200 mg/kg of EF were not significantly different (*P* > 0.05). When the antiplasmodial effects of these fractions were compared with the drug control (Artemether lumefanthrine), only the doses of HF and DF were as effective as AL on day 1. On day 3, in nearly all groups where there were interventions, had a significant difference in their parasite load when compared with the negative control but all doses of DF had the least parasite load. Similar results were obtained on day 5 (Table 1a). Consequently, this same fraction (DF) had the highest parasite clearance (Table 1b) when compared with the negative control. Throughout this experiment, there was a significant (*P* < 0.05) difference between parasite clearance in the test groups when compared with infected control. On day 1, there was no significant difference in percentage clearance between AL and DF. On day 5, the percentage parasite clearance of dichloromethane fraction of *D. mespiliformis* at 400 mg/kg was the highest among all test (fractions) groups.

**Table 1:** Mean percentage parasitemia in the blood of (a) mice treated with *D. mespiliformis* fractions and (b) experimental animals.

Treatments	Day 0	Day 1	Day 3	Day 5
**(a)**				
NC	1.6 ± 0.01	1.6 ± 0.04	6.0 ± 0.02	7.9 ± 0.01*
AL	6.0 ± 0.04	0.6 ± 0.03*	0.0 ± 0.00*	0.0 ± 0.00*
HF 100	0.8 ± 0.06	0.7 ± 0.08*	0.7 ± 0.10*	0.7 ± 0.05*
HF 200	0.6 ± 0.07	0.8 ± 0.02*	0.7 ± 0..02*	0.5 ± 0.06*
HF 400	0.6 ± 0.07	0.6 ± 0.01*	0.5 ± 0.02*	0.5 ± 0.02*
DF 100	1.4 ± 0.08	0.8 ± 0.06*	1.0 ± 0.02*	0.9 ± 0.02*
DF 200	1.6 ± 0.12	1.5 ± 0.06	0.7 ± 0.01*	0.4 ± 0.01*
DF 400	0.6 ± 0.08	0.4 ± 0.03*	0.4 ± 0.03*	0.4 ± 0.08*
EF 100	1.8 ± 0.02	1.5 ± 0.06	0.7 ± 0.08*	0.8 ± 0.02*
EF 200	1.8 ± 0.01	1.5 ± 0.10	1.2 ± 0.02*	1.2 ± 0.06*
EF 400	1.4 ± 0.00	1.1 ± 0.10*	0.8 ± 0.06*	0.8 ± 0.03*
MF 100	1.5 ± 0.03	0.8 ± 0.02*	1.5 ± 0.06*	1.8 ± 0.03*
MF 200	1.4 ± 0.04	1.1 ± 0.08*	0.8 ± 0.05*	1.2 ± 0.06*
MF 400	1.2 ± 0.06	1.0 ± 0.09*	0.8 ± 0.01*	0.8 ± 0.05*
**(b)**				
NC	0	0	0	0
AL	60 ± 0.02	70 ± 0.01	100 ± 0.00	100 ± 0.00
HF 100	50 ± 0.01	55 ± 0.06	60 ± 0.08	60 ± 0.12
HF 200	60 ± 0.21	70 ± 0.32	70 ± 0.46	65 ± 0.52
HF 400	60 ± 0.11	55 ± 0.07	60 ± 0.88	65 ± 0.21
DF 100	25 ± 0.21	65 ± 0.08	70 ± 0.06	70 ± 0.62
DF 200	30 ± 0.28	40 ± 0.52	70 ± 0.46	70 ± 0.07
DF 400	60 ± 0.42	40 ± 0.02	80 ± 0.05	80 ± 0.06
EF 100	10 ± 0.19	15 ± 0.06	35 ± 0.11	45 ± 0.52
EF 200	10 ± 0.08	15 ± 0.13	35 ± 0.42	50 ± 0.62
EF 400	25 ± 0.19	40 ± 0.51	45 ± 0.74	60 ± 0.75
MF 100	10 ± 0.09	40 ± 0.04	45 ± 0.63	45 ± 0.83
MF 200	25 ± 0.06	40 ± 0.72	45 ± 0.42	50 ± 0.32
MF 400	25 ± 0.28	50 ± 0.96	60 ± 1.26	60 ± 1.19

### Dichloromethane fraction of *D. mespiliformis* modulates hematological indices in infected mice

In view of the fact that malarial parasites invade the erythrocytes in its erythrocytic stage of infection, parasite load has a modulatory effect on the hematological parameter. The first blood cells infected are the erythrocytes. Erythrocyte infection affects the packed cell volume status of the host. In this study, the mean PCV of the negative control was the least (6%) while the PCV value for the DF group at 200 and 400 mg/kg were 25 and 20%, respectively. This is significantly (*P* < 0.05) higher than the untreated control. Interestingly, this mean PCV values of drug control (AL) and 200 mg DF/kg were not significantly different (*P* < 0.05) from the PCV value obtained from the normal group that were neither infected nor treated as shown in Table 2. Similar results were obtained for platelet counts except that 400 mg EF/kg platelet value was not significantly different from the normal control value. The WBC values however, were not significantly (*P* > 0.05) different when compared across the groups. Comparing the neutrophil count between the normal control and the test groups, we discovered that this blood cell value in the normal control were not significantly different (*P* > 0.05) when compared with AL and DF doses at 200 and 400 mg/kg, as well as EF at 400 mg/kg. Furthermore, the lymphocyte count of the normal control is significantly different from all test groups except 400 mg EF/kg, while both monocyte and eosinophil counts were not significantly different among the groups (Table 2).

**Table 2:** Mean haematological parameter of *P. berghei* infected mice after treatment with *D. mespiliformis* extract.

Treatments	PCV (%)	WBC (10^y^/μL)	Platelet (× 10^3^/L)	Neut (10^9^/μL)	Lym (10^9^/L)	Mono (10^9^/L)	Eosin (10^9^/L)
Nor	30 ± 1.30	10 ± 0.70	120 ± 6.80	72 ± 2.70	60 ± 4.70	5 ± 0.60	1 ± 0.00
NC	6 ± 0.20*	8 ± 0.10	105 ± 3.40*	50 ± 1.40*	50 ± 3.20*	6 ± 0.40	3 ± 0.00
AL	28 ± 0.10	10 ± 0.60	115 ± 3.90	70 ± 2.10	65 ± 2.80*	6 ± 1.20	1 ± 0.10
HF100	6 ± 0.10*	8 ± 0.20	80 ± 2.90*	58 ± 1.30*	68 ± 2.50*	6 ± 0.80	5 ± 0.80
HF200	10 ± 0.40*	9 ± 0.20	111 ± 2.90*	62 ± 1.60*	66 ± 1.50*	6Ú ± 0.60	6 ± 0.40
HF400	20 ± 1.20*	9 ± 0.60	112 ± 3.10*	60 ± 1.40*	68 ± 1.80*	7 ± 0.20	6 ± 0.60
DF100	8 ± 0.50*	10 ± 0.60	111 ± 6.50*	62 ± 1.70*	68 ± 1.50*	7 ± 0.50	7 ± 0.30
DF200	25 ± 0.30	10 ± 0.50	115 ± 4.20	70 ± 1.60	62 ± 1.50*	7 ± 0.40	6 ± 0.10
DF400	20 ± 0.20*	11 ± 0.80	111 ± 5.50*	65 ± 1.80	65 ± 1.60*	6 ± 1.60	7 ± 0.05
EF100	6 ± 0.20*	7 ± 0.05	70 ± 1.10*	50 ± 1.20*	50 ± 1.40*	6 ± 1.10	3 ± 0.15
EF200	8 ± 0.30*	10 ± 0.50	111 ± 5.40*	58 ± 1.10*	68 ± 1.60*	7 ± 0.20	6 ± 0.50
EF400	15 ± 1.50*	10 ± 0.40	115 ± 4.80	68 ± 1.80	65 ± 1.40	6 ± 1.80	5 ± 0.80
MF100	20 ± 0.40*	8 ± 0.70	112 ± 3.60*	60 ± 1.40*	70 ± 2.20*	8 ± 1.40	5 ± 0.40
MF200	15 ± 1.20*	10 ± 0.40	111 ± 3.20*	62 ± 1.50*	70 ± 1.80*	6 ± 0.80	5 ± 0.40
MF400	8 ± 0.60*	10 ± 0.80	111 ± 2.80*	62 ± 1.60*	68 ± 1.20*	7 ± 0.80	6 ± 0.60

Neut = Neutrophils, Lym = Lymphocytes, Mono = Monocytes and Eosin = Eosinophils. * = *P* < 0.05 normal control versus test groups.

### Dchloromethane fraction of *D. mespiliformis* has the least hemozoin content

**Figure 1:**
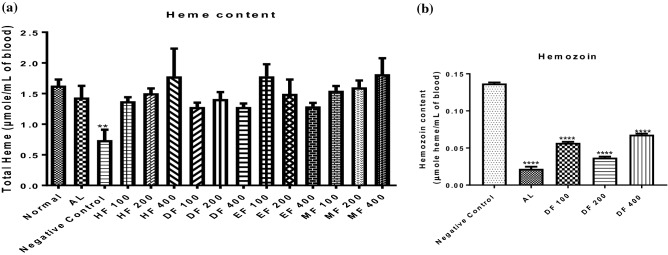
Heme (**a**) and hemozoin (**b**) contents of mice treated with fractions of *D. mespiliformis*. ** = *P* < 0.01 vs normal control in (**a**); **** = *P* < 0.0001 vs negative control in (**b**).

Although, the heme content of DF groups (100–400 mg/kg) is low compared to other fractions such as HF and MF at 400 mg/kg, this low level is not statistically significant when compared to the drug control group (Fig. 1a). Moreover, the heme content of the infected but untreated control decreased significantly (*P* < 0.01) when compared with the normal control. Since DF was the most potent fraction in the antiplasmodial activity study, we assayed only for this fraction’s hemozoin content. Comparing the level of hemozoin content between DF and untreated controls, it was observed that there was a significant (*P* < 0.05) decrease in hemozoin content of DF compared to the untreated control (Fig. 1b). This decrease was noticed maximally at 200 mg/kg, indicating that there was no linear correlation between the dose and decrease in hemozoin content.

### Solvent fractions of *D. mespiliformis* modulate mitochondrial permeability transition in mouse liver mitochondria

**Figure 2:**
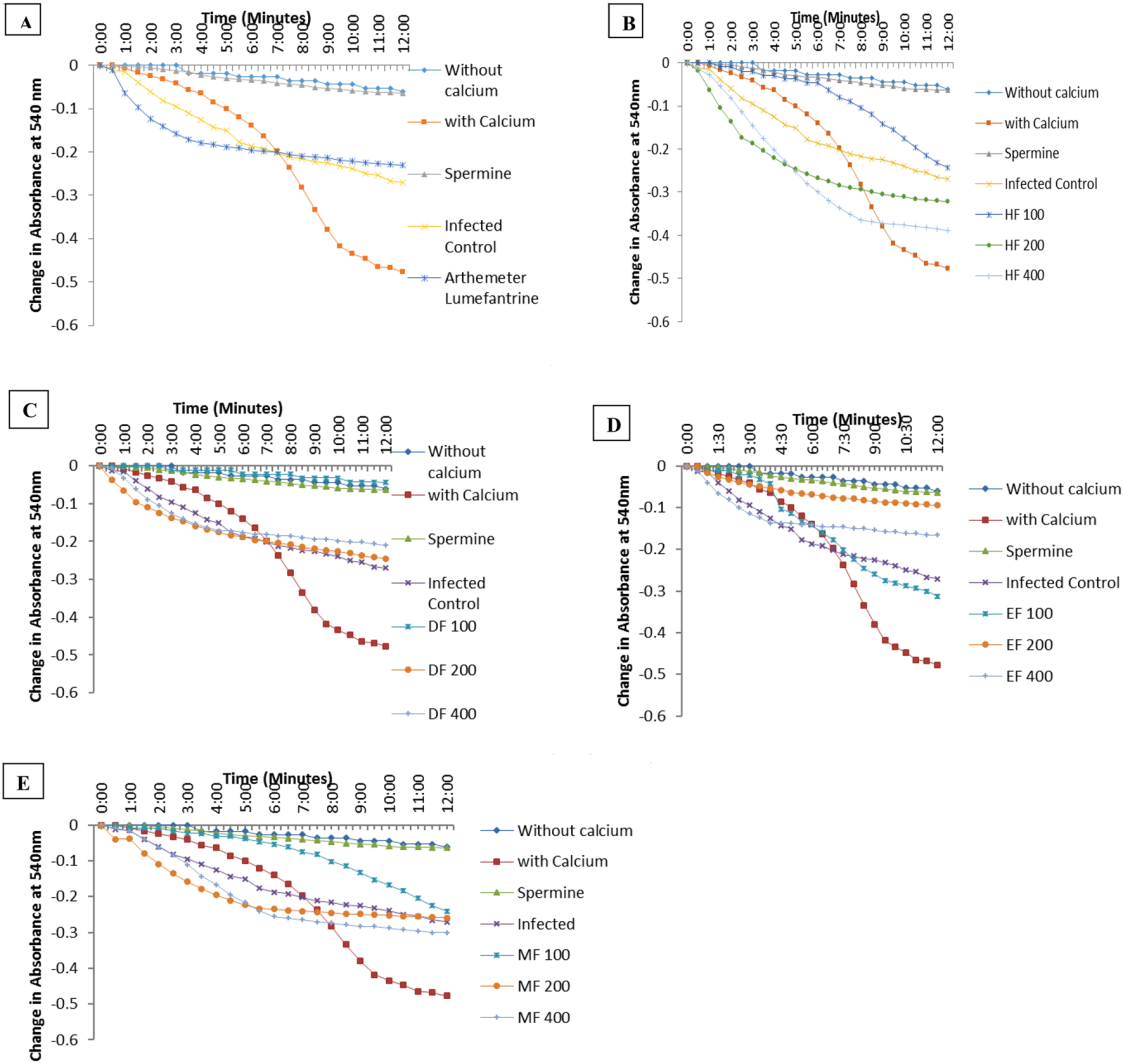
Representative profile of effects of *Diospyros mespiliformis* solvent fractions on mitochondrial permeability transition pore opening. (**A**) Isolated mitochondria integrity is shown by insiginifant decrease in changes in absorbance for 12 min at 30 s interval (without calcium), susceptibility of these mitochondria to calcium induction of the mPT pore opening was revealed by significant difference in changes in absorbance (withcalcium) and reversal by spermine was shown as insignificant decrease in changes in absorbance (spermine). (**B**) Hexane fraction (HF) of DM dose dependently opened the mPT pore maximally at 400 mg/kg and higher than infected control. (**C**) DF reversed opening of the pore by malarial infection and similar results were obtained in the EF group (**D**). (**E**) Only 400 mg/kg dose of MF opened the pore more than malarial infection.

Since the pre-erythrocytic stage of malarial infection occurs in the liver, we assessed the extent of the effect of malarial infection on hepatic mitochondrial permeability transition pore opening. We observed that malarial infection caused a large amplitude induction of the mitochondrial pore opening. Interestingly, artemether lumefantrine, the drug control, opened the mitochondrial pore more than malarial infection (Fig. 2A). Comparing the pore opening effects of all fractions of *D. mespiliformis*, we observed that HF opened the pore at 200 and 400 mg/kg, DF reversed opening of the pore maximally at 400 mg/kg, EF also reversed opening of the pore, while MF had a dose-dependent pore opening effect with a maximal effect at 400 mg/kg (Fig. 2B–E).

### Solvent fractions have minimal effects on mitchondrial F_1_F_0_ ATPase activity and peroxidation of membrane lipids

**Figure 3:**
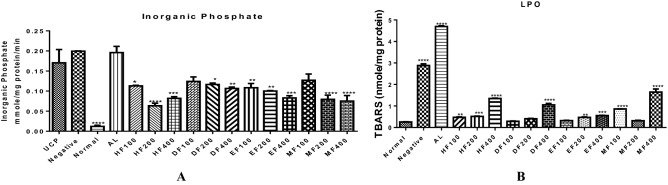
The ATPase activity of mitochondria isolated from the liver of experimental mice and mitochondrial lipid peroxidation results are presented in (**A**,**B**), respectively. Both infected control and control drug (Artemether lumefantrine) significantly (*P* < 0.001) enhanced ATPase activity while fractions of *Diospyros mespiliformis* significantly (*P* < 0.001) decreased ATPase activity (**A**). In (**B**), only DF 100, 200 mg/kg DF and 100 mg/kg EF were not significantly different from the normal control. The lipid peroxidation values of all groups were significantly lower than the negative control.

Host mitochondrial **F**_**1**_**F**_**0**_ ATPase activity was estimated to show the extent of inorganic phosphate generation via the enhancement of mitochondrial **F**_**1**_**F**_**0**_ ATPase. Our study showed that malarial infection caused a significant enhancement of mitochondrial **F**_**1**_**F**_**0**_ ATPase. However, all the fractions dose-dependently and significantly decreased the enhancement of mitochondrial **F**_**1**_**F**_**0**_ ATPase when compared with the infected control. It is interesting to note that the control drug (Artemether lumefantrine) enhanced ATPase activity (Fig. 3A). Malarial infection causes lipid peroxidation and so do some antimalarial drugs. We therefore assayed for mitochondrial **F**_**1**_**F**_**0**_ ATPase and lipid peroxidation to see the effects of the solvent fractions on these parameters. In Fig. 3B, all treated groups have significantly lower levels of TBARS compared with the negative control. The effects of these fractions on mitochondrial **F**_**1**_**F**_**0**_ ATPase showed untreated malarial infection (infected control) and AL enhanced mitochondrial **F**_**1**_**F**_**0**_ ATPase activity. Although all solvent fractions of *D. mespiliformis* enhanced mitochondrial ATPase activity when compared with the normal control, enhancement by fractions of *D. mespiliformis* were not statistically significant when compared with the control drug (Fig. 3A).

### Immunomodulatory effects of dichloromethane fraction of *D. mespiliformis*

**Figure 4:**
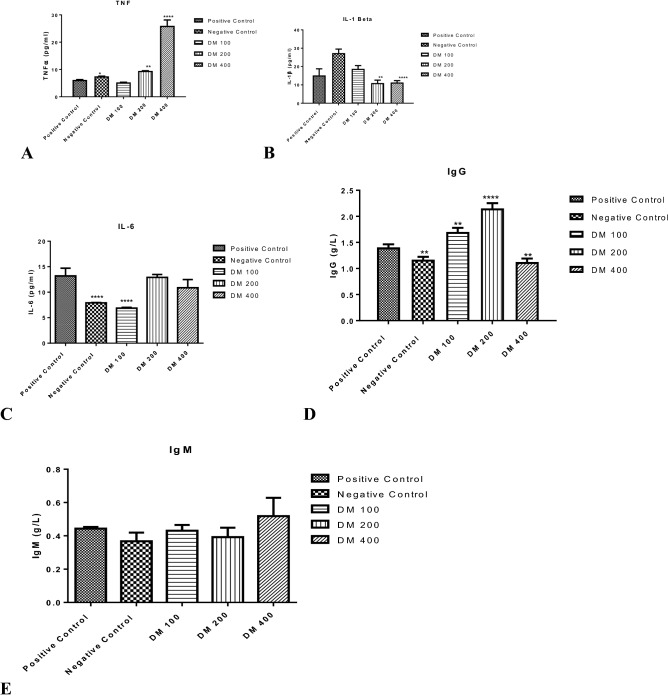
Immunomodulatory effects of *Plasmodium* infection and influence of treatment with dichloromethane fraction of *Diospyros mespiliformis*. (**A**) Serum TNFα significantly increased (*P* < 0.0001) maximally at 400 mg/kg of dichloromethane fraction of *D. mespiliformis* administration. (**B**) Serum interlukin (IL-1β) significantly (*P* < 0.0001) decreased in the DF-treated groups relative to the drug control while serum interlukin 6 (IL-6) of infected control and 100 mg/kg DF significantly (*P* < 0.0001) decreased relative to positive control (**C**). (**D**) IgG value increased maximally at 200 mg/kg of DF and (**E**) there is no significant difference in the IgM value in all the groups.

We also determined the influence of malarial infection on inflammatory cytokines and how treatment with DF modulated the serum concentrations of Tumour Necrosis Factor alpha (TNFα) and Interlukins 1 beta and 6 (IL-1*β* and IL-6).We observed that TNFα was dose-dependently higher (*P* < 0.001 at 400 mg/kg) compared with both normal and untreated control (Fig. 4A). However, the difference between the concentration of TNFα obtained in serum of infected mice treated with the control drug, artemether lumefanthrine (AL) and untreated infected mice were not significantly different (*P* > 0.05). In IL-1*β* however, the serum concentration of this cytokine was significantly (*P* < 0.001) higher in the AL-treated mice relative to other groups (Fig. 4B). The serum concentration of IL-6 in infected control and DM (100 mg/kg) were significantly lower compared with mice treated with AL and higher doses of DM (200 and 400 mg/kg). Moreover, the increase in IL-6 value did not linearly vary with doses of DM because the value of this cytokine significantly increased maximally at 200 mg/kg (Fig. 4C).

Furthermore, we determined the effect of malarial infection on immunoglobulins A and M (IgG and IgM, respectively) and the effects of curative intervention using graded doses of the dichloromethane fraction of *D. mespiliformis*. We observed that serum concentration of IgG decreased significantly in the infected control but increased in the AL-treated. The maximum increase was seen at the 200 mg/kg dose of the dichloromethane fraction of *D. mespiliformis* (Fig. 4D). There is however, no significant difference in the serum concentration of IgM in all groups (Fig. 4E).

### The GC–MS analysis of dichloromethane fraction of *D. mespiliformis*

**Figure 5:**
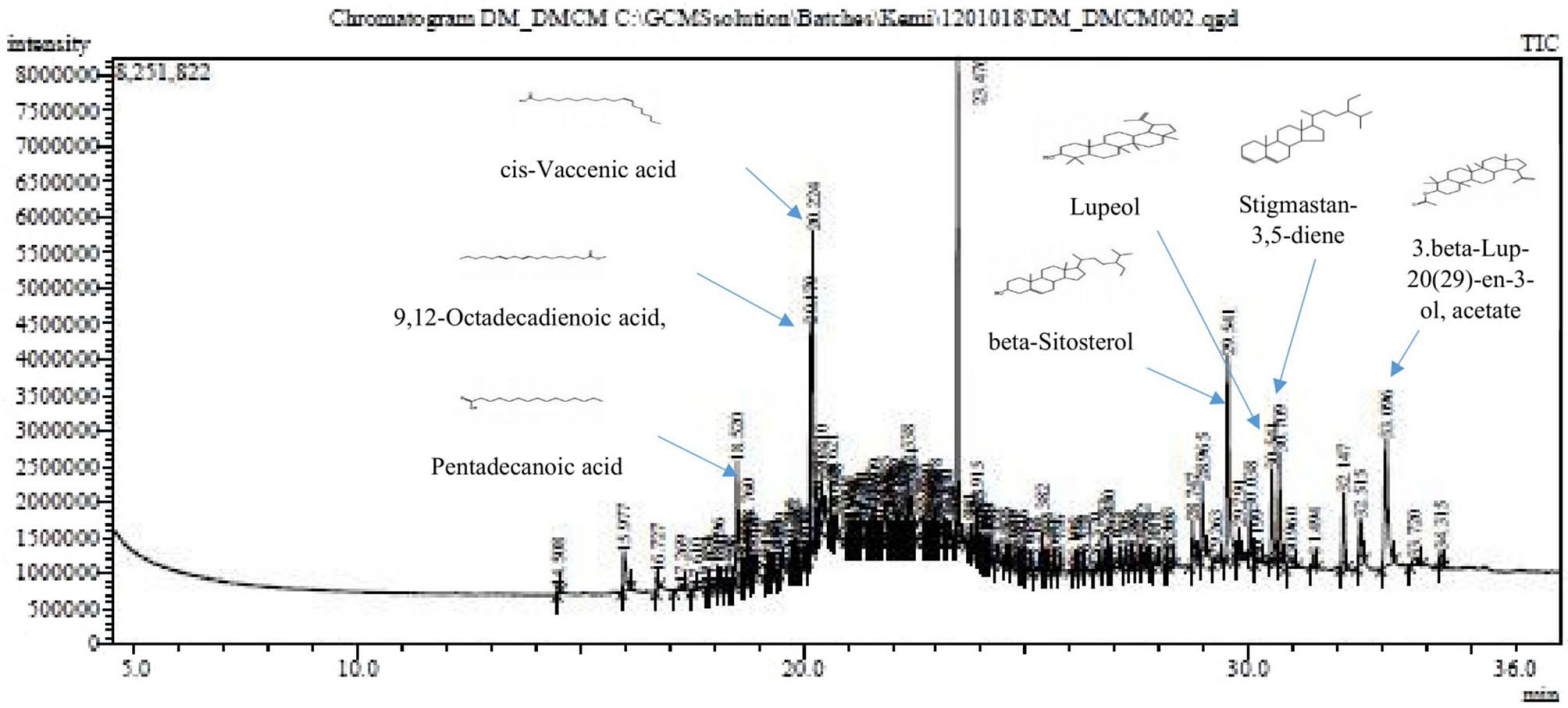
The GC–MS chromatogram of dichloromethane fraction of *D. mespiliformis.*

The GC–MS analysis of DF showed that many compounds are present in this fraction as represented in the chromatogram (Fig. 5). However, we observed that of all peaks that denote the presence of natural products in this fraction, there are approximately seven compounds that are most abundant. These are: pentadecanoic acid, octadecanoic acid methyl ester, *cis*-vaccenic acid, *β*-sitosterol, lupeol, stigmastan,3,5-diene and a lupeol derivative; 3*β*-lup-20(29)-en-3-ol acetate, arranged in order of decreasing abundance (Table 3).

**Table 3:** Most abundant compounds present in dichloromethane fraction of *Diospyros mespiliformis.*

S/N	Peak number	Height	Suggested name	Molecular weight	Formula	Suggested structure
1	11	18.520	Pentadecanoic acid	242	C_15_H_30_O_2_	
2	28	20.170	9,12-Octadecadienoic acid, methyl ester	294	C_19_H_34_O_2_	
3	29	20.224	cis-Vaccenic acid	282	C_18_H_34_O_2_	
4	110	29.541	beta-Sitosterol	414	C_29_H_50_O	
5	114	30.541	Lupeol	426	C_30_H_50_O	
6	115	30.709	Stigmastan-3,5-diene	396	C_29_H_48_	
7	120	33.096	3.beta-Lup-20(29)-en-3-ol, acetate	468	C_32_H_52_O_2_	

## Discussion

Malarial scourge is one of the worst infectious diseases worldwide. Although malaria parasites affect erythrocytes during the erythrocytic stage, the pre-erythrocytic stage in the liver leaves a deleterious effect on hepatocytes and its organelles, especially mitochondria. Mitochondrial pathology during malarial infection makes chemotherapy that clears the parasite insufficient for complete malarial treatment. Some antimalarial drugs cause more damage to host mitochondria and make them unsuitable for malarial treatment. Therefore, malarial drugs with a wide spectrum of anti-parasitic potential with concomitant protective effects on mitochondria and other organelles is highly desirable for malarial chemotherapy. In this study, we assessed the antimalarial potential, mito-protective effects and immunomodulatory properties of the dichloromethane fraction of the methanol extract of *D. mespiliformis.* The doses used in this study were appropriate since these were the doses used in the pilot study that showed antiplasmodial potential without toxic effects. Furthermore, our previously reported study^24^ showed that these doses were well tolerated and did not pose any toxic effect in experimental animals.

Using the established infection model, the dichloromethane fraction was discovered to have the highest parasite clearance and the least percentage parasitemia among all solvent fractions used. Although several studies have confirmed the antimalarial potential of *D. mespiliformis,* this is the first time an activity-guided assay on the antimalarial potential of this plant would be linked to a solvent fraction. Previous studies have shown that *D. mespiliformis* methanol extract contained terpenes, alkaloids, flavonoids and other phenolics that may have antimalarial properties^15^. Interestingly, the GC–MS analysis of this fraction showed the presence of pentadecanoic acid, 9,12-octadecadienoic acid methyl ester, *cis*-vaccenic acid, *β*-sitosterol, stigmastan-3,5-diene, lupeol and a lupeol derivative, 3*β*-llup-20(29)-en-3-ol acetate in appreciable quantity in addition to other natural products.

These compounds are likely to be major parts of the natural products responsible for the observed antimalarial activities of this fraction. It is also likely that these phytochemicals inhibit critical pathways such as folate biosynthesis in the parasite, causing oxidative stress in the organism or inhibiting polymerization of hemozoin to impact oxidative stress as their mechanisms of antiplasmodial potentials. Interestingly, the fatty acids and their methyl esters have been reported to have antimalarial properties by inhibiting fatty acid biosynthesis in *Plasmodium falciparum*^36^. Although, vaccenic acid has not been found to have antimalarial property, previous studies have shown that it can be converted to linoleic acid^37^ which has been reported to have strong antimalarial properties^35^. Further to this, *β*-sitosterol is ubiquitous in medicinal plants and has anti-inflammatory and antipyretic properties among others which are pathological events in malarial infection^38^. Stigmastan-3,5-diene was identified in this plant for the first time. It has been reported previously in other medicinal plants and reported to have antimicrobial properties^39^. This may offer a useful co-treatment effect for the treatment of microbial co-infection with malaria. Lupeol is a lupane-type triterpene with several medicinal properties. This compound and one of its derivatives, 3*β*-llup-20(29)-en-3-ol, have been identified in the dichloromethane fraction of *D. mespiliformis.* It is a pharmacologically active triterpene with antiprotozoal, anti-inflammatory, antimicrobial and chemo-preventive properties^40^. Furthermore, major complications of malarial infection occur as hepatic failure, because of the destruction of liver cells during the pre-erythrocytic stage of malarial infection causing jaundice and hepatomegaly. Hepato-protective lupeol can prevent this pathological events in malarial infection^41^. Similarly, renal protection by lupeol can ameliorate acute kidney injury that occurs in malarial infection^42,43^. Taken together, it appears these natural compounds are working together for the common purposes of protective and chemotherapeutic efficacies. This synergism arises due to the complex varieties of natural products individually or as potent mixtures of different classes of natural compounds for therapeutic purposes and for the prevention of pathological events in disease states. It is likely that medicinal plants have better medicinal effects than some orthodox drugs because of their multiple components for curative and ameliorative effects. This way, the side effects are minimized in addition to their antiplasmodial activity.

The study further confirmed that indeed, hematological abnormalities occur in malarial infection. This is due to a reduction in hemoglobin content, packed cell volume and red blood cell count. The malaria parasite multiplies and ingests its contents for survival in red blood cells. The invasion and multiplication of malaria parasites in red blood cells exert pressure on the erythrocyte cytoskeleton, damage hemoglobin contents via degradation and cause eventual burst of the red cell, further causing hemolysis, decrease in number of the red cells and anemia.. Anemia is one of the major complications in malarial infection. Although the pathogenesis of anemia in malaria may not be fully understood, it is however, clear that the destruction of red blood cells via parasite invasion, removal of both infected and non-infected red blood cells, and dyserythropoiesis via bone marrow dysfunction are all potential factors of anemia. Specifically, increase in parasite number via sexual and asexual reproduction coupled with oxidative stress puts pressure on the erythrocyte cytoskeleton leading to cell lysis and ultimate decrease in erythrocyte count. Furthermore, digestion and conversion of hosts’ cellular components for the parassite’s need makes eryptosis (cell death version in erythrocytes) inevitable, leading to a decrease in red blood cell count, anemia and a decrease in PCV. The significant increase in red blood cell, hemoglobin and packed cell volume is directly related to a significant decrease in total clearance of malaria parasites responsible for these pathological events. Furthermore, an increase in PCV, RBC count and hemoglobin as observed in this study when the solvent fractions of the plant were administered could be a result of hematopoietic influence and a proof of the potency of the administered fractions of *D. mespiliformis*^44^.

Leucopenia is a frequently observed complication in malarial infection. Specifically, lymphocytosis, monocytosis and decrease in neutrophil count are pathological manifestations of both severe and uncomplicated malaria^45^. In this study, WBC count and differentials in the treated groups increased significantly compared to the untreated group. Lymphocytes, neutrophils and monocytes are the centers for the biosynthesis of antibodies, chemokines and cytokines that may elicit immunological response during malarial parasite infection. A significant increase in neutrophils, monocytes and lymphocyte counts, as observed in this study, is beneficial for prevention against malarial parasite infection. Although erythrocytes are the first target of the parasites during the pre-erythrocytic stage, prolonged infection may further put pressure on other blood cells such as decrease in white blood cells (leucopenia) observed in the untreated infected control in this study. White blood cell differentials such as lymphocytes, neutrophils and monocytes are responsible for humoral and cell-mediated immunity during infection.

Lack of drugs or drug candidates that could decrease the parasite load in the infected control leads to a decrease in this inherent cellular defense. In both clinical and experimental malarial studies using human and murine models, phagocytes (monocytes and neutrophils) have been demonstrated to engulf both free parasites and parasite-infected red blood cells, thus decreasing parasite load^46^. It is interesting to note that thrombocytopenia is a major and common feature of pathology in malarial infection because of a significant decrease in platelets. Previous studies have further confirmed that a decrease in platelet count is observed in both mild and severe malaria infection^47^. In this study, treatment with DF of *D. mespiliformis* however, caused an increase in platelet count, which is not significant from what was observed in the animals in the group treated with the control drug. Biological functions of platelets in infection includes the maintenance of blood homeostasis and binding plasmodium-infected erythrocytes and killing the parasites within. This way, platelets has protective effect in malarial infection. The influence of leucocytes, monocytes and thrombocytes in malarial pathology and treatment reveals that host healing process involves several cellular reactions.

The heme molecule forms a major part of the red blood cell and is critical for oxygen transport for cellular respiration. During *Plasmodium* infection, heme degradation follows invasion of the erythrocytes and causes reduction in cellular aerobic respiratory processes. Because of erythrocyte destruction and its conversion to hemozoin, heme content decreases in malarial infection. The hemozoin content on the other hand, being a by-product of heme detoxification increases in untreated malaria. It is interesting to note that the dichloromethane fraction of *D. mespiliformis* increased heme content but significantly decreased hemozoin content. The observed increase in heme content in mice treated with dichloromethane fraction of *D. mespiliformis* further showed that this fraction decreased parasite load that could break down heme in the red blood cells and thus increase cellular respiration. Although the hemozoin molecule has frequently been described as a means by which the parasite evade the toxic heme and being a by-product of heme catabolism, its formation through heme breakdown would definitely indicate a decrease in the oxygen carrying capacity of erythrocytes and thus implies a decrease in cellular respiration. In this study, the DF of *D. mespiliformis* inhibited heme breakdown to hemozoin and ultimately would support aerobic respiration. Inhibition of heme polymerization to form hemozoin is a critical step for parasite survival in erythrocytes and has been targeted as a mechanism of action of antimalarial drugs.

Furthermore, it is interesting to note that hemozoin, a unique parasite metabolite also induces the production of a group of potent endogenous pyrogens including IL-1*β*^48^. By inference, a decrease in hemozoin content is expected to induce a decrease in the level of IL-1*β* as observed in this study.

High metabolic rate coupled with rapid growth and increase in parasite load are responsible for high levels of reactive oxygen species^49^. As a result of this imbalance in the ROS and inherent antioxidant system, the resultant oxidative stress is felt in mitochondria and other cellular organelles. Results obtained in this study have shown that DF of *D. mespiliformis* inhibited mitochondrial lipid peroxidation quantified using thiobarbituric acid reactive substances in mitochondrial isolates. It could be that phytochemicals present in the fraction have proton donating potentials that could neutralize the oxidants capable of causing peroxidation of mitochondrial membrane lipids. Reactive species are responsible for oxidative damage caused by an imbalance in the oxidant/antioxidant ratio. The observed decrease in these reactive species in infected mice treated with DF of *D. mespiliformis* is indicative of its antioxidant potential in addition to its antimalarial properties. Furthermore, malarial infection has been shown to be associated with mitochondrial pathology via the opening of the permeability transition (mPT) pore. Our previous report^50^_,_ in agreement with what is observed in this study shows that indeed, malarial infection causes mPT pore opening. Hexane, and methanol fractions of *D. mespiliformis* opened the mPT pore just as artemether lumefantrine (control drug) did. The unselective opening of the pore by antimalarial drugs poses a serious challenge to cell survival especially in un-infected cells. Interestingly, the DF fraction reversed mPT pore opening indicating that mitochondria as energy generating centers for the cells are preserved. It therefore follows that the integrity of mitochondria and their metabolic roles are preserved. We have previously provided evidence that some antimalarial drugs open the mPT pore leading to apoptosis^50^. In such case, it takes time for mitochondria to assume the full responsibility of their metabolic roles. Antimalarial drugs that inhibit pore opening in un-infected cells will enhance cell survival and prevent tissue wastage.

Many studies have been dedicated to malarial parasite mitochondrial ATPase^51^, while some studies have been dedicated to the parasite mitochondrial ATPase as a drug target^52^. Few studies have been carried out to determine what happens to host liver mitochondrial F_1_F_0_ ATPase during malarial infection and treatment with approved antimalarial drugs. Our previous studies have shown that indeed, mitochondrial F_1_F_0_ ATPase activity is enhanced in malarial infection and when treated with some drugs. We have also shown previously that there is a direct relationship between mitochondrial permeability transition pore opening and enhancement of liver mitochondrial F_1_F_0_ ATPase activity^53^. This is because the inorganic phosphate (Pi) released is an inducer of pore opening. Consequentially, the stimulation of mPT by inorganic phosphate absorbed into the cell or released via F_1_F_0_ ATPase activity has adverse effects on conservation of energy and indirectly on the survival of the cell via permeability transition^54^. The observed decrease in the activity of F_1_F_0_ ATPase favours a decrease in the cytosolic concentration of Pi through ATP hydrolysis and that could explain one of the reasons why mPT pore opening was reversed. Again, this could decrease bioenergetic dysfunction that is usually noticed in malarial infection and in situations where drugs used for the treatment of the disease caused enhancement of ATPase activity. Our observation that hepatic mitochondrial F_1_F_0_ ATPase activity decreased in mice treated with dichloromethane fraction of *D. mespiliformis* is an indication that ATP hydrolysis in the liver decreased, thus making it available to drive anabolic reactions.

Absolute clearance of malarial parasites from the host’s body depends on both adaptive and humoral immune systems. Although, there are contradictory data on the role of inflammatory cytokines in early protection against malarial infection, they play a significant role in protecting the host against malaria in the early stages of infection. It has been shown that although malarial infection is associated with high parasitemia which culminate in mortality, this condition is associated with a decrease in serum TNFα level^55^. In this case, an increase in serum TNFα level as provided by DF (at 400 mg/kg) observed in this study, is thought to offer protective effects against malarial infection. Interestingly, the increase in serum TNFα level noticed in this study was dose dependent. To further justify this finding, it has been discovered that TNFα can kill the malarial parasite, which can probably be mediated through NO production^56,57^. We therefore, infer that TNFα increase among others, may be one of the possible mechanisms responsible for the decreased percentage parasitemia at 400 mg/kg of DF.

During acute malarial infection, an increase in the cytokine level contributes to parasite clearance because of their curative potential. However, increase in these inflammatory cytokines may also indicate pathological conditions during malarial disease. In this study, we observed a significant decrease in IL-1*β* levels as the dose of DF increased, which probably correlate with parasite load. A scientific report has linked glycosylphosphatidylinositol from *Plasmodium* with the induction of IL-1*β*^58^.

Interlukin 6 is immediately produced in response to infection and tissue injury. Although the synthesis of this molecule is controlled at transcriptional and post transcriptional levels, dysregulated expression of IL-6 is known to lead to pathological events such as inflammation and autoimmunity^59^. Environmental stress and infection trigger the expression of IL-6 for host defence. Therefore, an increase in IL-6 level that was observed in this study is meant for the protection of the host cells against infection and tissue damage. Previous study has found correlation between decreased IL-6 level with thrombocytopenia^60^. We therefore, infer that increase in platelet count earlier observed in the treated groups in this study may be the reason why there was an increase in IL-6 levels which, we believe, might have worked synergistically to decrease parasite load and reduce disease progression. Previous evidence has shown that thrombocytes kill circulating parasites. This platelet-directed killing of *Plasmodium* species including *P.berghei* used in this study, is highly important in controlling the parasite load via the infected erythrocytes^61^. It is evident that if IL-6 upregulates the synthesis of thrombocytes, regulated increase in IL-6 level is beneficial in malarial therapy.

The quality and quantity of immune response in malarial parasite infection is of critical importance in the overall protection against malarial disease. Antibody response against infected erythrocytes is a critical step in providing adaptive immune response against malarial infection. This is possible via destruction of infected erythrocytes through eryptosis or phagocytosis of infected erythrocytes. Immunoglobulins, especially IgG and IgM are important antibodies that confer immunity against infection, especially the merozoite stage of the malarial parasite^62^. The increase in serum IgG level observed in this study when infected animals were treated with DF of *D. mespiliformis* showed that the phytochemical constituents present in this fraction is capable of increasing IgG levels, thus eliciting an immune response against malarial parasites.

Although serum Immunoglobulin M (IgM) level in the infected control is not significantly different in the treated groups (Fig. 4e), our observation that increase (albeit insignificant) in serum IgM both in the animals treated with standard drug and those treated with DF of *D. mespiliformis* showed that coupled with IgG, IgM also mediates in protective effects against parasite invasion of the host cell. Although, maintenance of IgM response may not follow a dose dependent pattern as observed in this study, its increase may have a very important immune response against primary and subsequent *P.berghei* infections. Taking together, increase in the circulating concentrations of IgG and IgM is beneficial for immediate parasite clearance and to confer immunity on the host for sometime.

In summary, our findings have suggested that the antimalarial mechanism of DF may be via its modulation of the inflammatory cytokines as related to malaria and may further have a protective role by upregulating the IgG and IgM levels. It is interesting to note that as against previous observation where an increase in TNF and IL-1 levels have shut down oxidative respiration in mitochondria, which may subsequently lead to mitochondrial pathogenesis, there may possibly be some counteracting processes that prevent this from happening^63–65^^.^ Our observation showed that this increase is beneficial. We further showed that although, the DF of *D. mespiliformis* significantly reduced parasite load and decreased the hemozoin content, it prevented mitochondrial pathology via the inhibition of mPT opening, thus maintaning ion homeostasis and preventing cellular damage and cell death in un-infected hepatocytes. This study further provides information on the possible mechanism of action of DF as an antimalarial recipe via its inhibition of hemozoin formation. Since the formation of hemozoin (malaria pigment) is one of the possible ways that the malarial parasite evades oxidative damage, inhibition of this process is a possible mechanism of action of DF, causing cellular damage to the organism and at the same time the host cells are prevented. Although, our results suggest possible mechanism of action of this fraction, an unequivocal mechanism of action of this fraction was not carried out. This is one of the limitations of this study. It would be interesting therefore, to further screen this fraction, or isolate the active principle and optimise where necessary this drug candidate and see if it can be formulated as a purified antimalarial recipe for use. Furthermore, the mechanism of action of the isolated compound with the highest antimalarial property can be carried out.
